# First Experience With the Triolifter, a Novel Device for Organ Fixation Used as a Heart Positioner in Cardiac Surgery

**DOI:** 10.7759/cureus.56461

**Published:** 2024-03-19

**Authors:** Yukio Umeda, Yukihiro Matsuno, Shohei Mitta, Shoji Yoshikawa

**Affiliations:** 1 Cardiovascular and Thoracic Surgery, Gifu Prefectural General Medical Center, Gifu, JPN; 2 Food and Nutritional Science, Toita Women’s College, Tokyo, JPN

**Keywords:** cardiac surgery, device for organ fixation, triolifter, heart positioner, coronary artery bypass grafting

## Abstract

We describe our first experience with the Triolifter (Fuji Systems, Yokohama, Japan) in cardiac surgery. The Triolifter is a less expensive, novel organ fixation device developed as a fixation indenter mainly for traction of the lung under video-assisted surgery and is now available in Japan.

An 84-year-old man diagnosed with unstable angina pectoris underwent emergency coronary artery bypass grafting (CABG) under cardiac arrest. Following the declamping of the aorta and the resumption of the beating heart, bleeding from the left anterior descending artery (LAD) anastomosis site was observed. The Triolifter was used as a heart positioner to expose the anastomosis site for hemostasis in the setting of an on-pump beating heart. Hemostasis of the posterior descending artery (PDA) anastomosis site could also be confirmed by traction of the right ventricular anterior wall using the Triolifter. It could be effectively and safely used with neither significant subepicardial hematoma nor epicardial injury.

In Japan, the Triolifter might be used as one of the insurance-covered devices in off-pump CABG in the future, but globally, it could also be used in on-pump CABG without hesitation because it is so inexpensive.

## Introduction

A variety of devices have been developed and marketed as heart positioners and stabilizers for off-pump coronary artery bypass surgery (OPCAB) [1-4]. While the safety and stability of those devices have been established to some extent, there is some concern about their cost because the reimbursement system in Japan sets a ceiling of 2,000 USD as an all-inclusive device fee for OPCAB. As for conventional coronary artery bypass grafting (CABG) with cardiac arrest or on-pump beating CABG, additional costs for such devices are not covered but are included in the procedure fee. Therefore, new devices that are not only safe and effective but also inexpensive enough to use without hesitation are desired.

The Triolifter (Fuji Systems, Yokohama, Japan) was developed primarily as a single-use organ fixation indenter for video-assisted pulmonary traction and has been available for clinical use in Japan since September 2023 [5]. The price of Triolifter is quite inexpensive, compared to 5%-14% of the heart positioners available in Japan.

We report here the first experience with the Triolifter used as a heart positioner in cardiac surgery.

## Case presentation

In November 2023, an 84-year-old man was referred to our hospital with unstable angina pectoris and an elevation of troponin I (6.0631 ng/mL). Emergency coronary artery angiography revealed 99% stenosis of the proximal left anterior descending artery (LAD) and chronic occlusion of the proximal right coronary artery (RCA). Following intra-aortic balloon pumping (IABP) implantation, the patient was referred to our department for CABG.

**Figure 1:**
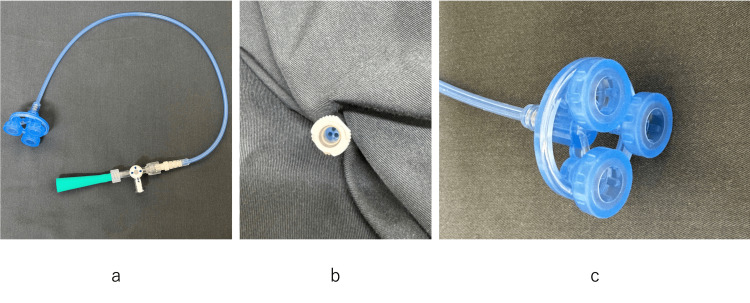
The Triolifter (a) The Triolifter manufactured by Fuji Systems, Inc., Yokohama City, Japan; (b) Three independent canals in the main tube; (c) Three cup-suckers are fixed circumferentially by a ring.

**Figure 2:**
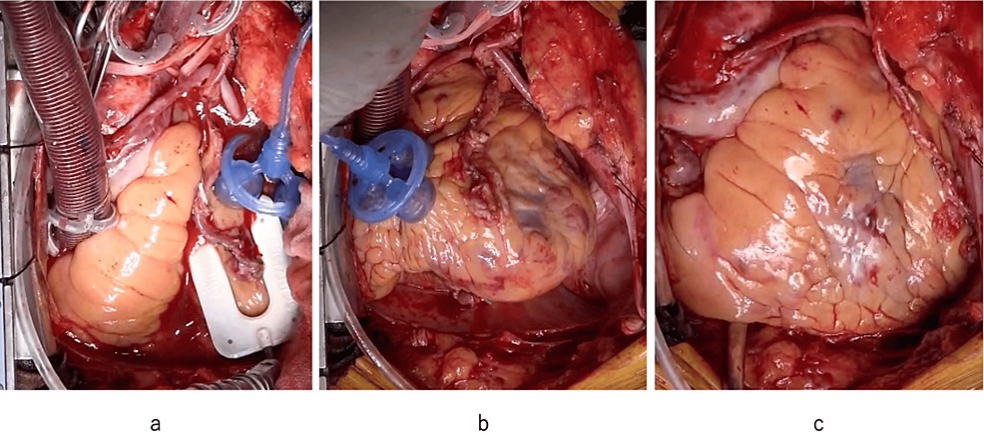
Triolifter used as a heart positioner (a) The left anterior descending artery (LAD) anastomosis site was exposed and stabilized by the Triolifter and the Assistant^TM^ attachment with StableSoft^TM^ technology; (b) The posterior descending artery (PDA) anastomosis site could be confirmed by traction of the right ventricular anterior wall using the Triolifter; (c) Neither significant subepicardial hematoma nor epicardial injury was observed at the suction site of the Triolifter.

Emergency CABG (left internal thoracic artery (LITA) grafting to LAD, aortic artery (AO)- saphenous vein graft (SVG) to posterior descending artery (PDA)) was carried out under cardiac arrest. After in-situ LITA to LAD anastomosis and SVG to PDA distal anastomosis, proximal anastomosis of SVG was performed at the ascending aorta after aortic root cannula removal. Following the declamping of the aorta and the resumption of the beating heart, bleeding from the LAD anastomosis site was observed. To avoid repetitive aortic clamp and cardiac arrest, we decided to add stitches for hemostasis under the on-pump beating heart. The Triolifter (Figure 1a) was applied to the anterior wall of the left ventricle to expose the LAD anastomosis site, and the Assistant^TM^ attachment with StableSoft^TM^ technology (two-finger, short, Terumo Corporation, Tokyo, Japan) was applied as a stabilizer. Then, an additional suture could be placed for hemostasis. (Figure 2a) After that, we also confirmed the hemostasis of the PDA anastomosis site by traction of the right ventricular anterior wall using the Triolifter (Figure 2b).

In this case, the Triolifter was used at a suction pressure of -60 kPa (-450 mmHg). The Triolifter was applied to the anterior wall of the left and right ventricles and attached stably and safely with no significant subepicardial hematoma or epicardial injury (Figure 2c). However, the three suction cup-suckers of the Triolifter were positioned relatively close, so they sometimes interfered with each other and did not fit well on non-planar surfaces, such as the apex or acute margin. Nevertheless, even when only two cups were attached to the heart, there seemed to be little decrease in suction force because of the independent long and narrow suction canals.

The post-operative clinical course of the patient was uneventful, with IABP weaning on post-operative day (POD) one, extubation on POD two, and discharge on POD 20. Patency of all grafts was confirmed by CT coronary angiography on POD seven. The patient is doing well four months after the operation. 

## Discussion

The Triolifter was developed as a single-use organ fixation indenter mainly for traction of the lung under video-assisted surgery and has been available for clinical use since September 2023 in Japan (Figure 1a). The Triolifter consists of three independent canals in the main tube connected to three cup-suckers through a rigid portion that can be grasped, and the three cups are fixed circumferentially by a ring (Figures 1b, 1c). Retraction can be achieved by pulling the tube or grasping the rigid portion with forceps. Since it was designed for video-assisted lung surgery, the three cups were connected with a relatively solid ring so that they could be inserted through the port by bending with the fingers as well as spread out in the thoracic cavity.

According to the analysis by Global Information, Inc., the global on-pump coronary artery bypass device (ONCAB) market is estimated to exceed $604.4 million by 2027. Also, the global OPCAB market is projected to reach $236.2 million by 2027. In their forecast period, the overall CABG market was considered to grow with a compound annual growth rate (CAGR) of nearly 7.8% [6]. On the other hand, increasing national medical care expenses is a major social problem in many countries. According to the Estimates of National Medical Care Expenditure of Japan, gross national medical expenses and medical expenses per capita are increasing year by year. The ratio of national medical expenses to national income has exceeded 10% since 2008 [7]. 

As the device market is projected to expand, there are concerns about its impact on the national healthcare system. Issues for hospital management, such as the ratio of costs to claimed medical expenses in individual cases, would be highlighted as well.

The reimbursement system in Japan actually sets a ceiling of 2,000 USD as an all-inclusive device fee for OPCAB, but the total price of stabilizers, heart positioners, proximal anastomosis devices, shunt tubes, and carbon dioxide (CO_2_) blowers exceed this ceiling. As for ONCAB, the reimbursement system sets no additional device fee, and the price of such devices used in the surgery is included in the procedure fee. These circumstances not only have a negative impact on hospital management but may also adversely affect clinical outcomes by discouraging the use of desirable devices for economic reasons.

**Figure 3:**
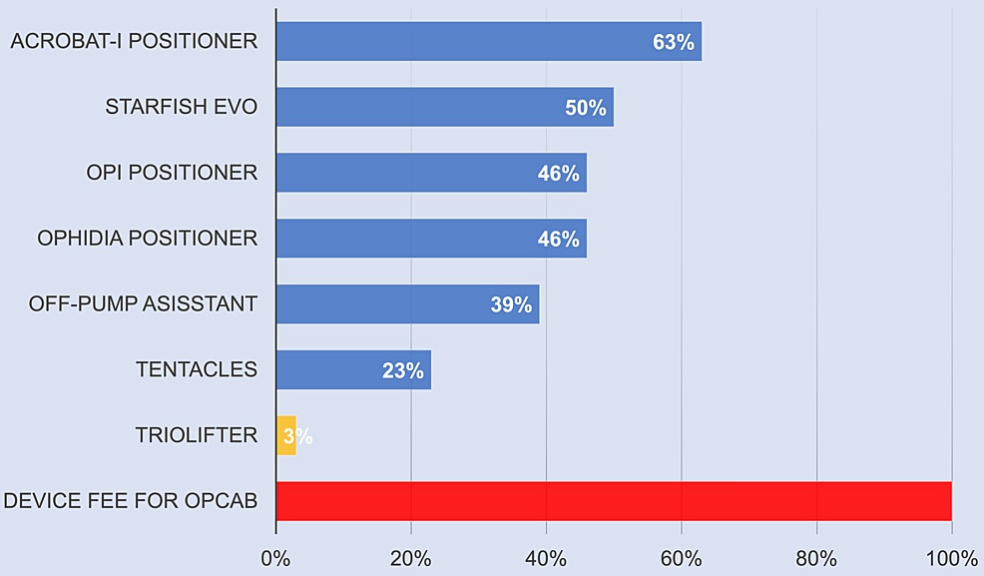
Percentage of each heart positioner's price for the all-inclusive device fee for OPCAB (2,000 USD) OPCAB: off-pump coronary artery bypass device

In fact, the use of heart positioners besides the Triolifter shown in Table 1 in ONCAB would account for 8%-21% of the procedure fee for CABG (8,590 USD), which is generally not acceptable considering the cost of the suture, aortic puncher, drainage tube, or cardiopulmonary bypass-related materials. On the other hand, the price of the Triolifter is inexpensive (67 USD), which is 5%-14% of the price of typical heart positioners, and it only accounts for 1% of the procedure fee for CABG. Also in the case of OPCAB, the Triolifter only accounts for 3% of the 2,000 USD all-inclusive device fee, while other heart positioners account for 23%-63% (Figure 3). Therefore, the Triolifter was considered easy to use in cardiac surgery without concern for cost. 

**Table 1:** Prices of heart positioners in Japan

Heart positioner	Company	Price	Reference
ACROBAT-i positioner	Getinge (Gothenburg, Sweden)	$1,260	[8]
Starfish Evo	Medtronic (Minneapolis, MN)	$1,000	[9]
OPI positioner	Mera (Tokyo, Japan)	$920	[10]
OPHIDIA positioner	Vital Corporation (Tokyo, Japan)	$920	[11]
OFF-PUMP Assistant	Terumo Corporation (Tokyo, Japan)	$784	[12]
Tentacles	SB-Kawasumi Laboratories, Inc (Akita, Japan)	$467	[13]
Triolifter	Fuji Systems (Yokohama City, Japan)	$67	[5]

Although the Triolifter would be expected to be used more frequently in OPCAB, we used it in the setting of an on-pump beating heart to confirm its safety and usability, as there were no reports of its use in cardiac surgery. In the present case, the Triolifter was stably used on flat surfaces but sometimes did not fit well on non-planar surfaces, such as the apex or acute margin. For use in cardiac surgery, the distance between the three cup-suckers might be slightly modified so that the cups do not interfere with each other to fit non-planar surfaces. The use of a heart positioner or stabilizer occasionally caused subepicardial hematoma or epicardial injury related to suction [14-15], but the use of the Triolifter at -60 kPa did not cause significant hematoma or injury.

In the future, with the extensive introduction of the Triolifter into ONCAB, further assurance of safety would be established by confirming the absence of physical damage such as subepicardial hematoma or epicardial injury through the accumulation of cases. On the other hand, when introducing the Triolifter into OPCAB, confirmation of the hemodynamic effects of the Triolifter on left- and right-ventricular function and mitral regurgitation should also be conducted in accordance with previous studies regarding pre-existing heart positioners [16-21].

In addition, heart positioners have been reported to be used during CABG for traction of the ascending aorta during proximal SVG anastomosis [22,23]. There are also reports of the utilization of heart positioners for cardiovascular surgery other than CABG, such as pericardium reconstruction after en bloc resection of mediastinal tumor [24], off-pump ablation for atrial fibrillation [25], and on-pump beating extra-anatomical ascending-descending aortic bypass [26]. The applicability of the Triolifter in these situations is the next issue to be discussed.

## Conclusions

In the setting of an on-pump beating heart, the Triolifter could be sufficiently used as a heart traction device for hemostatic maneuvers or to check an anastomosis site on the backside of the heart without obvious subepicardial hematoma or epicardial injury. Since the Triolifter is quite inexpensive and can be used without cost-related hesitation in a variety of countries with healthcare cost restrictions, its effectiveness should also be established in the off-pump setting through further accumulated use in coronary artery bypass surgery.
